# A Bayesian genome-wide linkage analysis of quantitative traits for rheumatoid arthritis via perfect sampling

**DOI:** 10.1186/1753-6561-1-s1-s110

**Published:** 2007-12-18

**Authors:** Cheongeun Oh

**Affiliations:** 1 Department of Preventive Medicine, University of Medicine and Dentistry of New Jersey, New Jersey 07101, USA

## Abstract

Rheumatoid arthritis is a complex disease caused by a combination of genetic, environmental, and hormonal factors, and their additive and/or non-additive effects. We performed a linkage analysis to provide evidence of rheumatoid factor IgM on linkage, based on Bayesian variable selection coupled with the new Haseman-Elston method. For statistical inferences to estimate unknown parameters, we utilized the perfect sampling algorithm, an emerging simulation technique that alleviates concerns over convergence and sampling mixing. Our methods provide powerful and conceptually simple approaches to simultaneous genome scans of main effects and all possible pairwise interactions. We apply them to the Genetic Analysis Workshop 15 data (Problem 2) provided by the North American Rheumatoid Arthritis Consortium (NARAC).

## Background

Rheumatoid arthritis (RA) is a clinically heterogeneous disorder with variability in severity, disease course, and response to therapy. Although the exact cause of rheumatoid arthritis is still unknown, RA is known to be a complex disease caused by a combination of genetic, environmental, and hormonal factors, and their additive and/or non-additive effects (epistases or gene × environment interactions). Genetic risk factors not only determine susceptibility for the disease but also correlate with disease severity and phenotype. Among phenotypes, rheumatoid factor IgM is a significant and common measure for diagnosis of RA. Therefore, genetic linkage analyses of IgM levels may reveal major differences in chromosomal regions showing evidence for linkage.

While recent interest has been focused on genome scans using a large number of marker loci, the common approaches of existing statistical methods produce often inconsistent results. This is due in part to the fact that they test markers one after another and fail to capture the substantial information of epistases among disease loci. The use of Bayesian model selection has been the popular method of remedying the pitfalls of conventional methods in recent years, whereby identifying loci with significant effects is viewed as a model selection problem. Unlike conventional methods suggesting a single best model, Bayesian methods consider multiple possible models along with their probabilities to incorporate model uncertainty. One of the powerful Bayesian model selection approaches is the use of stochastic search variable selection (SSVS) [1-4], in which Markov chain Monte Carlo (MCMC) sampling algorithms are used to sample from the posterior distributions, thus making identification of promising subsets even for many candidate variables (markers) feasible.

Although Bayesian approaches with MCMC techniques have made intensive computations possible and efficient on large-scale data sets arising in modern genomic and genetic applications, an application of Bayesian model selection is still quite challenging and limited from both a computational standpoint as well as the sensitivity to the choice of prior distributions. The usage of MCMC has been often controversial due to the uncertainty of convergence and the dependence on starting positions. In addition, the samples obtained by MCMC are correlated, which can drastically reduce the efficiency of the approaches. These drawbacks of MCMC, however, can be overcome by perfect sampling, which was first proposed by Propp and Wilson [5] under the name of coupling from the past (CFTP). Perfect sampling uses a scheme of coupling chains in order to guarantee that samples are exactly from the target distribution of interest. The basic idea is to run coupled chains that start from all initial states from the past time -*T *and run them to time 0, in which at any instant of time *t *∈ [-*T*, 0), the same random seed and an updating function are applied to all possible chains. Once all the chains meet (coalesce), from this time onward, due to the common random seed and an updating function, they follow the same path, and at a time 0 they arrive at the same state, which is then an exact sample from the posterior distribution. Therefore, this procedure guarantees that the effect of initial states wears off, yielding an exact sample regardless of starting values. Although perfect sampling suggests the ideal approach to draw an exact sample, the framework of running chains from all possible states is almost infeasible because of the large number of markers involved.

Motivated by Huang and Djuric [6], we propose an efficient implementation of perfect sampling for high-dimensional data. Then, coupled with the new Haseman-Elston method [7], we carry out screening to identify susceptibility alleles that are more closely linked to rheumatoid factor IgM. We further evaluate their possible epistases. Most existing methods adopt a two-stage procedure to screen epistases, in which epistases are only considered for previously selected markers with significant main effects, and thereby they are bound to miss important loci whose effects influence a trait primarily through epistasis. In contrast, we perform an efficient simultaneous screening both on main effects and epistases. Our methods can handle large problems involving up to thousands of markers without any strict conditions in a reasonable running time. We apply these methods to the RA data of Genetic Analysis Workshop 15 (GAW15) (Problem 2).

## Methods

### Haseman-Elston method

The simple regression method of the Haseman-Elston [8] offers an effective framework for studying linkage between markers and disease. Later, Elston et al. [7] proposed modifications to the original Haseman-Elston method [8] to improve its power. It is based on regression of the squared sum of mean-centered trait values, *CP*_*j *_= (*Y*_1*j *_- *m*)(*Y*_2*j *_- *m*), with mean *m *on the estimated proportion of alleles shared identically by decent (IBD) by the sibling pair, *Y*_1*j*_, *Y*_2*j*_.

### The model and prior specifications

Assume that there are *p *markers on the whole genome with *n *dependent data (samples). Then, we form a model that includes a number of different marker loci to study their simultaneous effects, which can be best approached in a linear regression fashion such as

$$\begin{array}{cc} y_{j}=\mu +\sum_{i=1}^{p}x_{ij}\beta_{i}+\varepsilon , & j=1,...,n, \end{array}$$

where *μ *is the mean, *y*_*j *_is an observed phenotypic value (*CP*_*j*_) for each sibling pair, *x*_*ij *_is a proportion of IBD for *i*^th ^marker in *j*^th ^sample, and *β*_*i *_is an effect of the *i*^th ^marker. The variance of the trait is assumed to be *ε *~ *N*(0, *φ*^-1^*I*), with *φ *being a precision parameter. To explore promising subsets (a set of markers having evidence of linkage) over the entire model space efficiently, a binary indicator *γ*_*i *_is used to represent an exclusion or inclusion of *i*^th ^marker in the model [1]. Then a model is represented by *γ *= (*γ*_1_,..., *γ*_*p*_) and Eq. (1) can be reduced to the *p*_*γ *_= *I*_*p*_*γ *variables by ignoring columns of *X *for which *γ*_*i *_= 0. We denote the corresponding model as *X*_*γ *_and coefficient parameters as *β*_*γ*_. When epistases are considered, the indicator vector *γ *is expressed as *γ *= (*γ*_1_,..., *γ*_*p*_, *γ*_(1, 2)_,..., *γ*_(*i*, *j*)_,..., *γ*_(*p*, *p*-1)_), where *γ*_(*i*, *j*) _is an indicator of an epistasis of *i*^th ^and *j*^th ^markers and Eq. (1) is extended by adding $x_{ij_{1}}x_{ij_{2}}\beta_{j_{1}j_{2}}$ for an epistatic effect, $\beta_{j_{1}j_{2}}$ between loci *j*_1 _and *j*_2 _≤ *p*. Therefore, a general model to describe both main effects and epistases can be written *Y *= *μ *+ *X*_*γ*_*β*_*γ *_+ *ε*, where some of the columns of *X*_*γ *_are formed from the original variables by multiplication of columns of *X *to build the design matrix for epistases and *β*_*γ *_= [*β*_1_,..., *β*_2_, *β*_1, 2_,..., *β*_*p*-1, *p*_].

The prior distribution for unknown parameters Φ_*γ *_= (*β*_*γ*_, *γ*, *φ*^-1^) can be decomposed as *π *(*β*_*γ*_, *γ*, *φ*^-1^) = *π *(*β*_*γ*_|*γ*, *φ*^-1^) *π *(*γ*) *π *(*φ*^-1^) under a simple independence assumption. We assume the prior Φ_*γ *_to be in the conjugate normal-gamma family, namely,

$$\begin{array}{cc} \pi (\beta_{\gamma },\gamma ,\varphi^{-1})=N_{p_{\gamma }}(0,c\varphi^{-1}I_{\gamma }), & \pi (\varphi^{-1})\propto \varphi^{-1}, \end{array}$$

where *c *is an unknown positive scalar.

### Posterior inference

The statistical inference for the identification of marker loci having evidence of linkage is retrieved through the posterior distribution of *γ*, which is given by Bayes' rule, *π *(*γ*|*Y*) ∝ *π *(*γ*)*f*(*Y*|*γ*). After nuisance parameters *β*_*γ *_and *φ*^-1^are integrated out from the marginalized likelihood, it is simplified to

$$\begin{array}{c} \pi (\gamma |Y)\propto \pi (\gamma )\int \int f(Y|\beta_{\gamma },\gamma ,\varphi^{-1})\pi (\beta_{\gamma }|\varphi^{-1},\gamma )\pi (\varphi^{-1})d\beta_{\gamma }d\varphi \\ =\pi (\gamma )\cdot \frac{{\left(Y^{T}Y-Y^{T}X_{\gamma }{\left(c^{-1}I_{P_{\gamma }}+X_{\gamma }^{T}X_{\gamma }\right)}^{-1}X_{\gamma }^{T}Y\right)}^{-(n-1)/2}}{{\left|1+cX_{\gamma }^{T}X_{\gamma }\right|}^{1/2}}. \end{array}$$

When there are a large number of markers involved, Eq. (2) is estimated via MCMC algorithms by simulating samples from the posterior distribution without knowing the normalizing constant, but at a risk of false inferences and being subject to initialization biases. We use perfect sampling described as follows.

### Posterior simulation via perfect sampling

Under a non-epistatic model, *γ *= (*γ*_1_, K, *γ*_*p*_), for example, we simulate samples from Eq. (2) by updating *γ *in a component-wise manner. Each component *γ*_*i *_is chosen consecutively or via a random permutation on its index (1,..., *p*). Then the probability of determining *γ*_*i *_to be 1 conditional on other latest updated components is given from a Bernoulli trial such as

$$P(\gamma_{i}=1|\cdot )=\frac{\pi (\gamma_{\left(i\right)})f(Y|\gamma_{\left(i\right)})}{\pi (\gamma_{\left(-i\right)})f(Y|\gamma_{\left(-i\right)})+\pi (\gamma_{\left(i\right)})f(Y|\gamma_{\left(i\right)})},$$

where *γ*_(-*i*) _= (*γ*_1_,...., *γ*_*i*-1_, *γ*_*i*+1_,..., *γ*_*p*_) and *γ*_(*i*) _= (*γ*_1_,...., *γ*_*i*-1_, 1, *γ*_*i*+1_,..., *γ*_*p*_). There are 2^*p*-1 ^possible configurations of Eq. (3). The original perfect sampling method, CFTP [5], entails running parallel chains from every possible 2^*p*-1 ^state from the past time -*T *to 0 repeatedly until it achieves coalescence. However, our approaches do not require running all these chains based on two following ideas.

First, for *t *∈ [-*T*, 0), instead of attempting to run all possible chains, we construct, *sandwich distributions*, which bound all the possibilities of Eq. (3) such as

$$L_{i}^{t}\leq P(\gamma_{i}^{t}=1|\cdot )\leq U_{i}^{t},$$

so that an update is done only on these two distributions. This is because the coupling of these sandwich distributions implies the coalescence of all other chains in between. Second, rather than tracing $\gamma^{t}=(\gamma_{1}^{t},\ldots ,\gamma_{p}^{t})$, we generate its support $S^{t}=(s_{1}^{t},\ldots ,s_{p}^{t})$ to keep track of only possible values, which further reduce the computational burden. That is, for a random seed $u_{i}^{t}$ generated from a uniform distribution on (0, 1), if $L_{i}^{t}\geq u_{i}^{t}(U_{i}^{t}\leq u_{i}^{t}),\gamma_{i}^{t}=1(\gamma_{i}^{t}=0)$ is taken as true and its support $s_{i}^{t}$ is assigned as the same value. On the other hand, if $L_{i}^{t}\leq u_{i}^{t}\leq U_{i}^{t},\gamma_{i}^{t}$ is indeterminate and $s_{i}^{t}$ records uncertain values, {0, 1}. Then, for all *i *= 1, 2,..., *p*, an updating rule is formulated as

$$s_{i}^{t}=\{\begin{array}{ll} \left\{0\right\} & \text{if }u_{i}^{t}\geq U_{i}^{t}\\ \left\{1\right\} & \text{if }u_{i}^{t}\leq L_{i}^{t}\\ \left\{0,1\right\} & \text{otherwise} \end{array}.$$

Coalescence is achieved when all supports become settled at time 0, i.e., |$s_{i}^{t}$| = 0 for all *i*. This procedure in implemented iteratively as follows. At *T *= -1, for each $s_{i}^{t}$ ∈ *S*^*t*^, we decide two sandwich distributions and update *S*^0 ^based on Eq. (5). If the coalescence is achieved, a support *S*^0 ^is reported as a draw from the target posterior in Eq. (2). Otherwise, we move back at *T *= -2 and repeat updating for *t *∈ [-2, 0], and then check coalescence at *0*. The whole procedure is repeated, and a sample is drawn only if coalescence occurs at *0*. Otherwise, the starting time is shifted further back, preferably at -2*T *[5] and the updates perform by reusing the same random seed, which is critical to preclude the space from growing [5]. One of the main keys in our methods is to construct two bounds, $L_{i}^{t}$ and $U_{i}^{t}$. We have recently proposed how to build these bounds, approximately to succeed the perfect sampling even for high dimensional spaces. The manuscript may be obtained upon request.

### Model space prior for epsitases

To account for epistatic effects, we consider two different model space priors of *π *(*γ*). An independence prior is usually used when it is believed that effects of markers influence the trait entirely independently of each other. In this case, we have

$$\pi (\gamma )=\prod_{j=1}^{p}\pi (\gamma_{i})\prod_{i=1<j}^{p}\pi (\gamma_{\left(i,j\right)})=\left(\prod w_{1}^{\gamma_{i}}{\left(1-w_{1}\right)}^{1-\gamma_{i}}\right)\left(\prod w_{2}^{\gamma_{\left(i,j\right)}}{\left(1-w_{2}\right)}^{1-\gamma_{\left(i,j\right)}}\right),$$

where *w*_1 _and *w*_2 _are hyper-priors for the inclusion of main effects and epistases, respectively. It is reasonable to choose that *w*_2 _≤ *w*_1 _≤ 0.5. Alternatively, we can embed the dependent structure of main effects and epistases [9] such as

$$\pi (\gamma )=\prod_{j=1}^{p}\pi (\gamma_{i})\prod_{i=1<j}^{p}\pi (\gamma_{\left(i,j\right)}|\gamma_{i},\gamma_{j}),$$

where the conditional probability for an epistasis to be included, *γ*_(*i*, *j*) _= 1 takes on four different values depending on the main effects,

$$\pi (\gamma_{\left(i,j\right)}=1|\gamma_{i},\gamma_{j})=\{\begin{array}{ll} w_{00} & \text{if (}\gamma_{i},\gamma_{j})=(0,0)\\ w_{01} & \text{if (}\gamma_{i},\gamma_{j})=(0,1)\text{ or (}\gamma_{i},\gamma_{j})=(1,0)\\ w_{11} & \text{if (}\gamma_{i},\gamma_{j})=(1,1) \end{array}.$$

This dependent relationship can be advantageous in that we can reduce the size of the model space by limiting certain epistases to be included in the model. For example, if we believe that an epistasis should be considered only if at least one of the main effects is significant, we let *w*_00 _= 0. However, because we might miss important marker loci that might affect a phenotype primarily through epistasis, it may be more reasonable to have 0 ≤ *w*_00 _≤ *w*_01 _≤ *w*_11 _≤ 0.5. The hyper-priors, (*w*_1_, *w*_2_) in Eq. (6) and (*w*_11_, *w*_01_, *w*_00_) in Eq. (7), can indirectly control the expected numbers of effects in the model. Therefore, small values are essential because we expect there are a small number of markers linked to the trait.

### Selection criterion

After we collect samples using perfect sampling, the identification of markers that are tightly linked to the genes is given by estimates of marginal posterior probabilities. To this end, we simply count the relative frequencies of model visits in the samples, and the marginal posterior of the *i*^th ^marker being important is estimated by summing over the posterior of models containing this marker. Then, we list the estimates of marginal posterior probabilities in a numerical order. Their patterns are used to gauge the importance of effects. When the decision is made, the model space prior (*w*_1_, *w*_2_) in Eq. (6) and (*w*_11_, *w*_01_, *w*_00_) in Eq. (7) play an important role as threshold values. If the marginal posterior probability of the marker is higher than these values, we decide that the corresponding effect of this marker is significant.

### Data

We used rheumatoid factor IgM as the quantitative trait values and microsatellite scans for 511 multiplex families over the 22 autosomal chromosomes. The IBD values were obtained using the statistical software MERLIN [10]. A total of 590 independent sib pairs and 407 microsatellite markers were used in the analysis.

Our programs were written in MATLAB and each was run on Super Macintosh G5 with a 2.66 GHz quad-processor.

## Results

### Choice of hyper-parameters

Before we ran perfect sampling, we had to decide hyper-parameters (*c*, *w*_1_) under a non-epistatic model, and (*c*, *w*_1_, *w*_2_) in Eq. (6) or (*c*, *w*_11_, *w*_01_, *w*_00_) in Eq. (7) under an epistatic model. To specify these values, rescaling and sensitivity analysis may be advisable. We checked the sensitivity of our methods toward the choice of *c *by re-running our algorithms for several values of *c *between 1 and 10. The results were not sensitive (data not shown). Choosing (*w*_1_, *w*_2_) and (*w*_11_, *w*_01_, *w*_00_) in the model space prior is rather straightforward. A smaller value should provide smaller estimates of marginal posteriors, but our results were robust to these values since we took them as threshold values to select important effects. Therefore, in this paper, we only reported the results by fixing (*c*, *w*_1_) = (5, 0.01) for a non-epistatic model and (*c*, *w*_1_, *w*_2_) = (5, 0.01, 0.01) in Eq. (6) and (*c*, *w*_11_, *w*_10_, *w*_00_) = (5, 0.01, 0.01, 0.005) in Eq. (7) for an epistatic model for comparison.

### Main effects

**Figure 1:**
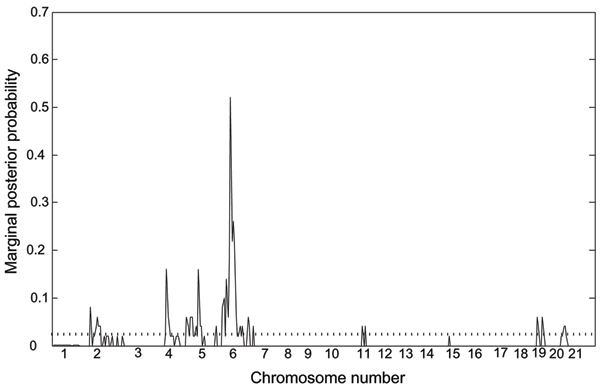
**Marginal posterior probabilities of component *λ***. The highest peak on chromosome 6, and suggestive peaks on chromosomes 2, 4, 5, 11, 19, and 21. All had estimated marginal probabilities higher than the model prior *w *= 0.01 (dotted line).

We first performed screening of main effects only. We collected 500 samples from perfect sampling. The average coupling time to achieve coalescence for one sample was about 1 minute. Figure 1 displays an empirical frequency of each effect to estimate a marginal posterior probability, *π *(λ_*i *_= 1|*Y*). We found the highest peak on chromosome 6, and suggestive peaks on chromosomes 2, 4, 5, 11, 19, and 21, which had estimated marginal probabilities greater than *w*_1 _= 0.01.

### Total effects (main and interaction effects)

To assess the evidence for epsitases, we included all main effects and two-way pairwise interaction terms in the model. Therefore, the total number of effects considered was 83,028. We compared two different assumptions Eq. (6) and Eq. (7). We collected 500 samples. The average coupling time to achieve coalescence for one sample was about 25 minutes under Eq. (6) and 21 minutes under Eq. (7). The summary of the results is given in Table 1. The same significant main effects as in the above "main effects" were found. Additionally, we found three suggestive interactions effects between chromosomes 6 and 16, 6 and 19, and 6 and 21 under both Eq. (6) and Eq. (7) prior assumptions.

**Table 1:** Ranking of empirical estimations of marginal posterior probability of significant effects under two prior assumptions

(*w*1, *w*2) = (0.01, 0.01)			(*w*_1_, *w*_11_, *w*_10_, *w*_00_) = (0.01, 0.01, 0.01, 0.005)		
	
Ranking	Chromosome	Relative frequency^a^	Ranking	Chromosome	Relative frequency
1	Chr 6	0.35	1	Chr 6	0.37
2	Chr 5	0.28	2	Chr 4	0.35
3	Chr 4	0.21	3	Chr 5	0.28
4	Chr19	0.17	4	Chr19	0.11
5	Chr 6 × Chr 19^b^	0.15	5	Chr 2	0.08
6	Chr 2	0.09	6	Chr 6 × Chr 16	0.05
7	Chr 6 × Chr 16	0.05	7	Chr 6 × Chr 21	0.04
8	Chr 6 × Chr 21	0.03	8	Chr 6 × Chr 19	0.02
	Others	<0.01		Others	<0.005

^a^Relative frequency corresponds to the frequency appeared in the samples. Effects that appeared more than once are displayed.

^b^"A × B" stands for an epistasis between chromosomes A and B.

## Conclusion

We have applied Bayesian variable selection via perfect sampling to the RA data of GAW15 to identify markers linked to rheumatoid factor IgM. Our methods can accommodate a large number of markers, permit epistatic effects to be considered in the models, and evaluate all effects simultaneously. Therefore, they have significant advantages over the classic approaches. As opposed to other Bayesian methods, our methods do not require any tunings relating to convergence issues of MCMC techniques and there is no dependence on initial values. Therefore, they are reliable even from a small number of drawn samples.

Our analyses have revealed that there is a strong evidence for main effects on chromosome 6, and also marginal evidence for epistases between chromosomes 6 and 16, 6 and 19, and 6 and 21. To increase the accuracy, we may collect more samples.

## Competing interests

The author(s) declare that they have no competing interests.
